# Intratarsal Keratinous Cyst Clinically Misdiagnosed as a Chalazion

**DOI:** 10.3390/dermatopathology11020014

**Published:** 2024-04-19

**Authors:** John Lennon Silva Cunha, Clenia E. S. Andrade, Fernando A. P. da Cunha Filho, Alexandre R. da Paz, Manuel A. Gordón-Núñez, Pollianna M. Alves, Cassiano F. W. Nonaka

**Affiliations:** 1 Postgraduate Program in Dentistry, Department of Dentistry, State University of Paraíba (UEPB), Campina Grande 58429-600, PB, Brazil; godonnunez152631@servidor.uepb.edu.br (M.A.G.-N.); cassiano.nonaka@servidor.uepb.edu.br (C.F.W.N.); 2 Residency Program in Oral and Maxillofacial Surgery, School of Public Health of Paraíba, João Pessoa 58040-440, PB, Brazil; 3 Department of Physiology and Pathology, Federal University of Paraíba (UFPB), João Pessoa 58051-900, PB, Brazil

**Keywords:** intratarsal keratinous cyst, chalazion, eyelids, dermatopathology

## Abstract

The intratarsal keratinous cyst (IKC) is a recently described entity, often clinically misdiagnosed as a chalazion. We report a case of a 61-year-old male patient with a chief complaint of a small lesion on the upper eyelid that evolved over six months. On physical examination, an asymptomatic, firm nodule was identified on the left upper eyelid. The patient reported no history of trauma. A provisional diagnosis of chalazion was established, and an excisional biopsy was performed. Histopathologically, the lesion was lined with a stratified squamous epithelium, with a corrugated epithelial surface showing abrupt keratinization without keratohyalin granules, and compact keratinous-appearing material in the cystic lumen. The diagnosis was IKC. No signs of recurrence were observed after one year of follow-up. It is essential to accurately diagnose IKC and distinguish it from chalazion and epidermal inclusion cysts, because IKC requires complete surgical excision and can exhibit multiple recurrences if not properly removed.

## 1. Introduction

The intratarsal keratinous cyst (IKC) is an entity recently described by Jakobiec et al. (2010) and often mislabeled in literature as “intratarsal epidermal inclusion cysts” [1]. The pathogenesis of the IKC has not yet been fully elucidated [2,3]. However, current evidence suggests that it originates from the meibomian glands, based on the similarity of the immunohistochemical profile observed between these glands and the cyst [4]. The true incidence of IKCs is also unknown. However, it has been suggested that these lesions represent the third leading cause of tarsal-related pathologies, after chalazia and sebaceous gland carcinoma [3,5].

Clinically, the lesions present as a small, non-inflammatory nodule, firm upon palpation and fixed to the tarsus, but with freely movable overlying skin on the upper eyelids of middle-aged male adults [3,6]. Although rare, multiple IKCs associated with the Gorlin–Goltz syndrome (GGS) have been reported [6]. Due to their non-specific clinical appearance, IKCs are often misdiagnosed as other eyelid lesions, particularly epidermal inclusion cysts and chalazia. Therefore, it is essential to diagnose the IKCs accurately and distinguish them from these lesions, because the IKC recurs locally if not completely removed [3,7,8]. Surgical excision of the cyst and the associated gland has been proposed to prevent recurrences [3]. On the other hand, the chalazion presents an indolent clinical behavior with no tendency to recur after conservative surgical excision [3,7,8].

Herein, we report an additional case of IKC in the left upper eyelid of a 69-year-old male patient, clinically misdiagnosed as a chalazion. Also, we reviewed the literature to provide a basis for a better understanding of this unusual lesion.

## 2. Case Report

**Figure 1:**
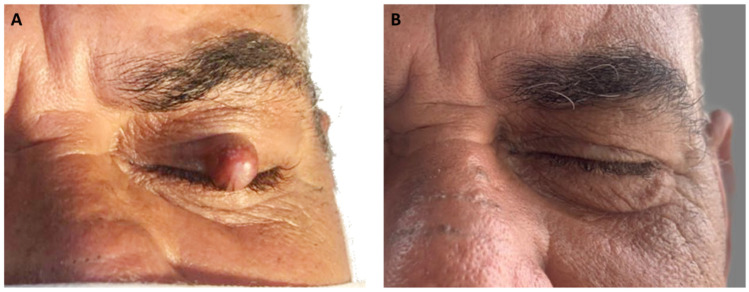
Clinical aspects of the intratarsal keratinous cyst. (**A**) Observe a well-delimited small nodular sessile lesion with a reddish color on the left upper eyelid. (**B**) Clinical appearance one year after lesion removal.

A 61-year-old non-white patient presented at an oral and maxillofacial medicine service with a six-month history of a lesion on the eyelid. Physical examination revealed a painless firm nodule on the left upper eyelid, measuring approximately 1.0 cm in diameter. No history of local trauma was reported (Figure 1A). The overlying skin was normal, with no inflammatory signs, and freely movable. Eversion of the eyelid revealed a subsurface intratarsal lesion with a slightly pale yellow bulging. The patient also complained of blurred vision in the left eye. A provisional diagnosis of chalazion was given, and total excision of the lesion through a lid crease incision was carried out.

**Figure 2:**
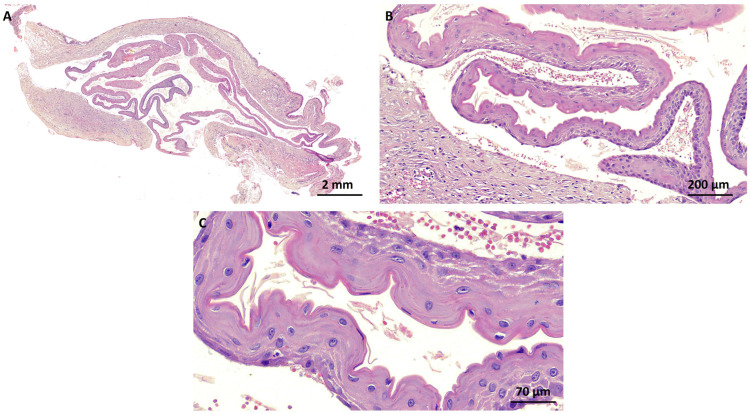
Histopathological aspects of the intratarsal keratinous cyst. (**A**,**B**) The cyst was lined with a keratinized squamous epithelium, composed of four–five layers of polygonal keratinocytes with a corrugated epithelial surface showing abrupt keratinization without keratohyalin granules. The epithelium–connective tissue interface was flat, and the cystic lumen was filled with abundant, eosinophilic, refractile, and string-like keratin. The cystic capsule was composed of poorly vascularized, tightly woven bundles of tarsal collagen with no evident inflammatory infiltrate. (**C**) Detail of the cystic epithelial lining displaying parakeratinized stratified squamous epithelium with a corrugated surface (hematoxylin and eosin stain).

Gross examination revealed a small, well-demarcated cystic cavity ranging in color from gray to brown, filled with a milky fluid material. Some hemorrhagic brownish regions at the periphery of the specimen were also observed. Histologically, the cyst was lined by a keratinized squamous epithelium, composed of four–five layers of polygonal keratinocytes with a corrugated epithelial surface showing abrupt keratinization without keratohyalin granules. The epithelium–connective tissue interface was flat, and the cystic lumen was filled with abundant, eosinophilic, refractile, and string-like keratin. The cystic capsule was composed of poorly vascularized, tightly woven bundles of tarsal collagen with no evident inflammatory infiltrate. No sebaceous glands were found on the cyst wall (Figure 2). The final diagnosis was IKC. No signs of recurrence were observed after one year of follow-up (Figure 1B).

## 3. Discussion

The term IKC was first introduced in 2010 by Jakobiec et al. to describe cysts whose histopathological appearance was similar to “epidermal inclusion cysts” [1]. Since then, a few well-documented cases of IKCs have been reported in the literature, most of them single case reports or small case series [1,2,3,6,7,9,10].

Clinically, IKCs present as a non-mobile submucosal nodule under the skin or conjunctiva of the upper eyelid [3,5,6], similar to the present case. IKCs are often solitary lesions (95%) and occur mainly in the upper eyelids (95%), with no significant gender difference, and typically manifest at an average age of 56 years [6]. The time of evolution is variable, ranging from a few months to years. Some cases have been associated with GGS [6]. In these circumstances, IKCs tend to be multiple (80%), occur bilaterally, and emerge at an earlier age (mean age 40) [6]. Only two cases of multiple IKCs (non-syndromic) have been reported [7,10]. However, the possibility that these patients are carriers of non-diagnosed GGS cannot be discounted, because both individuals were young, and one of them had a concomitant history of multiple facial basal cell carcinomas [7,10].

Clinically differentiating IKCs from other palpebral entities, especially chalazion, can be challenging [3]. However, some authors suggested that clinical and transurgical aspects can help this distinction [3,5]. The skin overlying the lesion has been described as slightly paler than the surrounding skin [5]. Upon palpation, a well-defined edge sensation with an acute angle between cyst and tarsus suggests an IKC that superficially involves the tarsal plate [5]. Blue-whitish or white coloration under the palpebral conjunctiva upon eyelid eversion indicates a deep location of the lesion, possibly within the tarsal plate [5]. Furthermore, the gross aspect of the IKC has the consistency of tofu residue or milky liquid, unlike the chalazion, which contains sebum and cellular debris [3,7]. However, although these findings suggest an IKC, the definitive diagnosis can only be made after histopathological analysis.

Furthermore, it is important to consider other lesions in the differential diagnosis, such as epidermoid cysts, pilomatricomas, and early sebaceous carcinoma [1]. Although these lesions may have some overlapping features, there are subtle differences that require thorough evaluation. Epidermoid cysts generally manifest as mobile, well-defined, fluctuating nodules, unlike the firm, fixed nature of IKCs [1,11]. Pilomatricomas often exhibit a characteristic “tent sign” on palpation due to calcification within the tumor, in contrast to the smooth surface typically associated with IKCs [1]. Furthermore, sebaceous carcinomas (meibomian) deserve special attention, due to their aggressive local behavior and potential for distant metastasis. In the early stages, it is challenging to differentiate them from other eyelid lesions due to clinical similarity. They generally present as slow-growing nodules, with irregular edges, and with the possibility of ulceration and bleeding, especially in areas exposed to the sun [12]. Understanding these nuances facilitates the establishment of a more accurate differential diagnosis, guiding the choice of appropriate therapeutic interventions and optimizing patient outcomes. However, histopathological examination remains essential for the definitive diagnosis.

Histologically, IKCs are lined by a corrugated keratinized stratified squamous epithelium with an apical cuticular surface and a characteristic immunophenotypic profile that supports the hypothesis that the origin of IKC is from dilated ducts of the meibomian ducts/tarsal plate [1,13]. Although the morphological differential diagnosis of dermal cysts is broad, the diagnostic possibilities are limited when the lesions are located on the upper eyelid and fixed to the tarsus. An important differential diagnosis of IKC is steatocystoma, possibly derived from the pilosebaceous glands [13]. However, steatocystomas usually present as multiple skin cysts (steatocystoma multiplex) or, less commonly, as a solitary cyst (steatocystoma simplex) in younger individuals (<30 years), unlike individuals with IKCs, who have a much older mean age [12,14,15]. Typically, steatocystoma contains yellow fluid and microscopic findings show a partially collapsed, thin-walled cyst lined with a stratified squamous epithelium, without a granular layer but with a thick, undulating eosinophilic cuticle and sebaceous glands in or near the wall [16]. Although there are notable similarities between IKCs and steatocystomas, it is possible to distinguish them through the presence of sebocytes in the lining epithelium and/or surrounding sebaceous glands (seen only in steatocystomas). Furthermore, the consistency of the keratin can vary, being typically laminar and with a “milky fluid” appearance in IKCs, while in steatocystomas it is thicker and yellowish in color [3,7,13].

Although the diagnosis is histopathological, immunohistochemical analysis allows the differentiation of IKCs from steatocystomas [13]. IKCs express CEA (carcinoembryonic antigen) and are positive for CK17 (indicating the presence of keratin type 1), EMA (epithelial membrane antigen) and CK19 (human keratin 19) [1,13]. On the other hand, steatocystomas generally do not express CEA, show weak or absent staining for CK19, and have positive staining for CK5 [13]. The absence of staining for CK17 in the superficial epithelium suggests the diagnosis of steatocystoma [13].

Although simple surgical excision is the most suitable treatment for common epidermoid cysts and steatocystomas, total tarsectomy has been recommended for IKCs of the meibomian glands to prevent recurrences [3,5]. The intratarsal location of IKCs is the main factor contributing to a higher recurrence rate, compared to other eyelid cysts. This position within the tarsal plate makes complete excision difficult, increasing the risk of leaving residual tissue, which in turn increases the likelihood of recurrence [1,13]. However, there was no difference reported between total or partial tarsectomy regarding the recurrence rates in reported cases [1,2,3,5,7,9]. Currently, surgical excision is performed either by the transcutaneous approach or by the transconjunctival approach [3,7]. A comparison between transconjunctival access and transcutaneous access for the treatment of IKCs also does not seem to influence the rate of recurrence or cosmetic problems [3,7]. Therefore, regardless of the surgical technique selected, the most important thing is to ensure the complete removal of the lesion.

Cases of IKC treated with cortisone injections into the cyst have been reported, but with unfavorable outcomes [9,13]. Finally, an amniotic membrane patch graft has also been suggested as an option to cover the surgical defect after surgery, and it has shown promising results [15]. In the present case, an anterior palpebral sulcus surgical approach with total intratarsal cyst excision and partial tarsectomy effectively prevented local recurrence. This procedure avoids more complex eyelid reconstructions. No signs of recurrence were observed after one year of follow-up.

## 4. Conclusions

In summary, the differentiation of IKCs from chalazia and other eyelid lesions is essential for proper surgical management and requires a detailed history, careful clinical evaluation, and palpation of the lesion with eyelid eversion. An accurate histopathological assessment is critical to avoid diagnostic errors and provide adequate management that involves complete excision of the lesion to prevent recurrences. A precise description of the lesion and the appropriate use of terminology are essential for a better understanding of these entities.

## Data Availability

The original contributions presented in the study are included in the article, further inquiries can be directed to the corresponding authors.
